# Dpb11 may function with RPA and DNA to initiate DNA replication

**DOI:** 10.1371/journal.pone.0177147

**Published:** 2017-05-03

**Authors:** Irina Bruck, Nalini Dhingra, Matthew P. Martinez, Daniel L. Kaplan

**Affiliations:** Florida State University College of Medicine, Department of Biomedical Sciences, Tallahassee, Florida, United States of America; Saint Louis University, UNITED STATES

## Abstract

Dpb11 is required for the initiation of DNA replication in budding yeast. We found that Dpb11 binds tightly to single-stranded DNA (ssDNA) or branched DNA structures, while its human homolog, TopBP1, binds tightly to branched-DNA structures. We also found that Dpb11 binds stably to CDK-phosphorylated RPA, the eukaryotic ssDNA binding protein, in the presence of branched DNA. A Dpb11 mutant specifically defective for DNA binding did not exhibit tight binding to RPA in the presence of DNA, suggesting that Dpb11-interaction with DNA may promote the recruitment of RPA to melted DNA. We then characterized a mutant of Dpb11 that is specifically defective in DNA binding in budding yeast cells. Expression of *dpb11-m1*,*2*,*3*,*5*,Δ*C* results in a substantial decrease in RPA recruitment to origins, suggesting that Dpb11 interaction with DNA may be required for RPA recruitment to origins. Expression of *dpb11-m1*,*2*,*3*,*5*,Δ*C* also results in diminished GINS interaction with Mcm2-7 during S phase, while Cdc45 interaction with Mcm2-7 is like wild-type. The reduced GINS interaction with Mcm2-7 may be an indirect consequence of diminished origin melting. We propose that the tight interaction between Dpb11, CDK-phosphorylated RPA, and branched-DNA may be required for the essential function of stabilizing melted origin DNA *in vivo*. We also propose an alternative model, wherein Dpb11-DNA interaction is required for some other function in DNA replication initiation, such as helicase activation.

## Introduction

DNA replication initiates in S phase with the activation of the replication fork helicase, which unwinds DNA at the front of an advancing replication fork [1, 2]. Central to activation of the replication fork helicase is the assembly of Mcm2-7 heterohexameric complex with the critical accessory factors, Cdc45 and GINS [2, 3]. The Cdc45-Mcm2-7-GINS complex (CMG) is the active replication fork helicase that unwinds DNA at a replication fork during S phase [3–6]. Recent structural data with budding yeast proteins indicates that the CMG encircles a single-strand of DNA as it unwinds double-stranded DNA [7]. Thus, CMG unwinds DNA by a steric exclusion mechanism [7]. The steric exclusion mechanism is also consistent with functional data from *Xenopus* extracts [8].

The Mcm2-7 is the motor of the CMG, because this heterohexameric complex is an active ATPase in the presence of Cdc45 and GINS [3, 9–11]. Mcm2-7 loads as a double hexamer to encircle double-stranded DNA during G_1_ phase [12, 13]. During S phase, the Mcm2-7 ring opens, and a single-strand of DNA is extruded from the central channel of Mcm2-7 [7, 8]. This process of strand extrusion is critical for activation of the replication fork helicase, since the CMG unwinds DNA by a steric exclusion mechanism [7, 8]. The extrusion of single-stranded DNA from the central channel of Mcm2-7 is also known as origin melting, and the mechanism for origin melting during S phase is currently unknown. However, it is generally considered that after origin melting, the melted origin ssDNA is coated with the eukaryotic single-stranded DNA binding protein, RPA [1].

Cdc45 is recruited to Mcm2-7 during S phase in a manner that depends upon the Dbf4-dependent kinase (DDK), a kinase that is specifically active during S phase [14–17]. Cdc45 recruitment to Mcm2-7 is also dependent upon Sld3, a protein required for replication initiation that does not travel with the replication fork [14, 18]. Sld3 recruitment of Cdc45 to Mcm2-7 has been shown to be dependent upon DDK phosphorylation of Mcm4 and Mcm6 [14].

**Fig 1:**
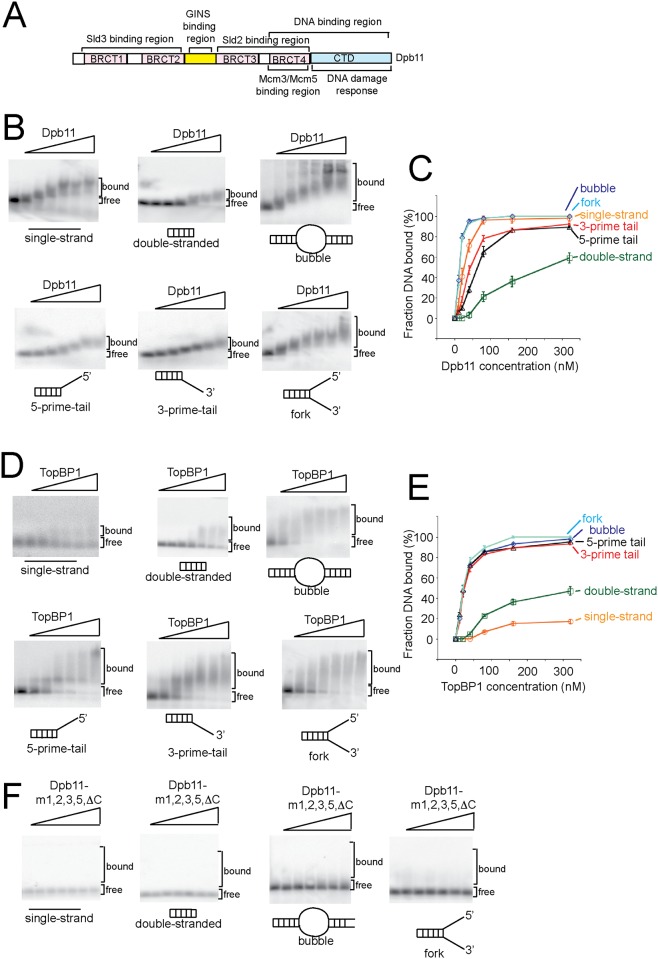
Yeast Dpb11 has high affinity for ssDNA or branched DNA structures, while human TopBP1 has high affinity for branched-DNA structures. (A) Schematic view of Dpb11 interaction with known binding partners. (B) Increasing concentrations of wild-type Dpb11 (0, 10, 20, 40, 80, 160, and 320 nM) were incubated with different DNA structures as described in Materials and Methods. The DNA sequences are detailed in Table 1; the DNA was radiolabeled with ^32^P. The products were analyzed by native agarose gel electrophoresis, and the dried gel was exposed to a phosphorimaging screen. (C) Experiments similar to those described in (B) were quantified, averaged, and plotted. The fraction DNA bound was plotted as a function of Dpb11 concentration. (D) Similar to (B), except human TopBP1 was used instead of budding yeast Dpb11. Identical DNA structures and protein concentrations were used as in (B). (E) Experiments similar to those described in (D) were quantified, averaged, and plotted. The fraction DNA bound was plotted as a function of Dpb11 concentration. (F) Similar to (B), except a mutant of Dpb11 (Dpb11-m1,2,3,5,*Δ*C) was used instead of wild-type Dpb11.

The recruitment of GINS to Mcm2-7 is also required for complete replication fork helicase assembly [19]. The assembly of GINS with Mcm2-7 is dependent on the S-phase cyclin-dependent kinase (S-CDK), a second kinase that is essential and specific for S phase [15]. S-CDK phosphorylates Sld3 and Sld2 (an additional essential initiation factor), and phosphorylated Sld2 and Sld3 bind to Dpb11 via its BRCT motifs (Fig 1A) [20, 21]. Thus, S-CDK promotes the formation of the Sld2-Sld3-Dpb11 ternary complex [20, 21]. In addition, Dpb11 binds to GINS via the region of the protein that lies between BRCT motif 2 and BRCT motif 3 (Fig 1) [22]. Dpb11 interaction with GINS is required for GINS assembly with Mcm2-7 *in vivo* [22].

We previously found that the Dpb11 binds to single-stranded DNA (ssDNA) using a GST-pulldown assay [23]. In addition, we found that the BRCT 4 motif of Dpb11, and the non-essential C-terminal domain of Dpb11 bind to single-stranded DNA [23]. Additionally, we found that a quadruple mutation of the BRCT4 motif of Dpb11 (m1,2,3,5, or dpb11-K463E, K469E, R538E, K549E), when combined with a deletion of the C-terminus of Dpb11 (deletion of amino acids to 616 to 764), results in a protein (Dpb11-m1,2,3,5-ΔC) with background binding affinity for ssDNA, as determined by the GST-pulldown assay [23]. This DNA-binding defective mutant of Dpb11 binds to Mcm2-7, Cdc45, Mcm2-7, Sld3, Sld2, and GINS like wild-type Dpb11, suggesting that the mutant we identified is specifically defective for binding DNA ([23]). We also previously observed that expression of *dpb11-m1*,*2*,*3*,*5* in a *dpb11-td* degron strain under restrictive conditions resulted in defective cell growth, defective DNA replication, and defective helicase assembly *in vivo* [23]. However, we have not yet characterized the phenotype for the Dpb11-DNA binding mutant, *dpb11-m1*,*2*,*3*,*5-ΔC*, in budding yeast cells.

In this manuscript, we characterize the interaction between Dpb11, DNA, and RPA *in vitro* and *in vivo*. *In vitro*, we find that Dpb11 binds to single-stranded DNA or branched DNA structures with high affinity. The human homolog of Dpb11, TopBP1, has a high affinity only for branched-DNA structures, suggesting a mechanism for binding to melted origin DNA. We also find that Dpb11 binds to CDK-phosphorylated RPA in the presence of branched DNA. Furthermore, we investigate the *in vivo* phenotype for the *dpb11-m1*,*2*,*3*,*5-ΔC* mutant, and we find that expression of this mutant in budding yeast cells results in a substantial decrease of RPA and GINS recruitment to a replication origin. These data suggest that Dpb11-DNA-RPA interaction may be required for origin melting in budding yeast cells. We also propose an alternative model, wherein Dpb11-DNA interaction is required for some other function in DNA replication initiation, such as helicase activation.

## Materials and methods

### Antibodies

Antibodies directed against RPA were purchased from Pierce, and antibodies against TopBP1 were purchased from Abcam. Antibodies directed against Mcm2, Cdc45, GINS, and Dpb11 were validated as described [23–25].

### Yeast strains

Degron strain *dpb11-td* [YJT70, MAT a ade2-1 ura3-1 his 3–11, 15 trp 1–1 leu2-3, 112 can 1–100 dpb11-td (DPB11 5’upstream -100 to -1 is replaced with kanMX-tTA tetR-VP16-tetO_2_-Ub-DHFRts-HA-linker) UBR1::GAL-Ubiquitin-M-lacI fragment-Myc-UBR1 (HIS3) leu2-3,112::pCM244 (tetR’-SSN6, LEU2)] was a generous gift from John F.X. Diffley (London Research Institute, Cancer Research UK, London, UK [20]). The degron strain *dpb11-td* was transformed with a PRS416 vector containing an empty vector, *DPB11* wild type, *dpb11-m1*,*m2*,*m3*,*m5*, *dpb11-ΔC*, *dpb11-m1*,*m2*,*m3*,*m5-ΔC*, under the control of native *DPB11* promoter. Positive transformants were selected on CSM-Ura plates.

### Plasmids and protein purification

The vectors for overexpression of human TopBP1 (full-length and fragments) were generously provided by Aziz Sancar as described [26]. Human TopBP1 was purified as described [26]. Plasmids for *E*. *coli* recombinant expression of Dpb11 (wild-type and mutant, GST- and PKA-) for PKA-GINS, and for yeast *in vivo* expression of *DPB11* (wild-type and mutants) were prepared as described [23, 27, 28]. Yeast Dpb11 (Wild-type and mutant), and GINS were purified as described [23, 25, 29, 30]. Briefly, Dpb11 (Wild-type and mutant) and GINS were each purified by nickel column chromatography, followed by Source Q purification, and finally gel filtration. Purification of GST-Dpb11 uses sequential nickel and glutathione resins. GST alone was purified as described [28]. Protein Kinase A was a generous gift from Susan Taylor (University of California at San Diego, San Diego, CA).

### Radiolabeling DNA

Single stranded DNA (ssDNA) was end labeled with T4 polynucleotide kinase (NEB), and radiolabeled ssDNA was purified over G-25 Sephadex Columns for Radiolabeled DNA Purification (Roche). To make double stranded DNA substrates (dsDNA) with radiolabeled ssDNA, 4 μl of 500 nM radiolabeled ssDNA was incubated with 4 μl of 500 nM complementary DNA in 4 μl of reaction buffer (20 mM Tris HCl, 4% glycerol, 0.1 mM EDTA, 40 μg/ml BSA, 5 mM DTT and 10 mM magnesium acetate) in a final volume of 12 μl. The reaction was incubated overnight at 37°C. The reaction was then diluted with 20 mM Tris-HCl, 0.1 mM EDTA to a final volume of 40 nM radiolabeled DNA.

### Kinase labeling of proteins

PKA and CDK-labeling of proteins was performed as described [29–31]. Proteins containing a PKA tag at the N-terminus were radiolabeled in a reaction volume of 100 μl that contained 20 mM PKA-tagged protein in kinase reaction buffer (5 mM Tris-HCl, pH 8.5, 10 mM MgCl_2_, 1 mM DTT, 500 μM ATP, 500 μCi [gamma-^32^P]-ATP containing 5 mg PKA. Reactions were incubated for 1 hour at 30°C. The kinase was then removed from the mixture by affinity chromatography. Complete removal of PKA or CDK protein was determined by Western analysis.

### Electrophoretic mobility sift assay (EMSA)

**Fig 2:**
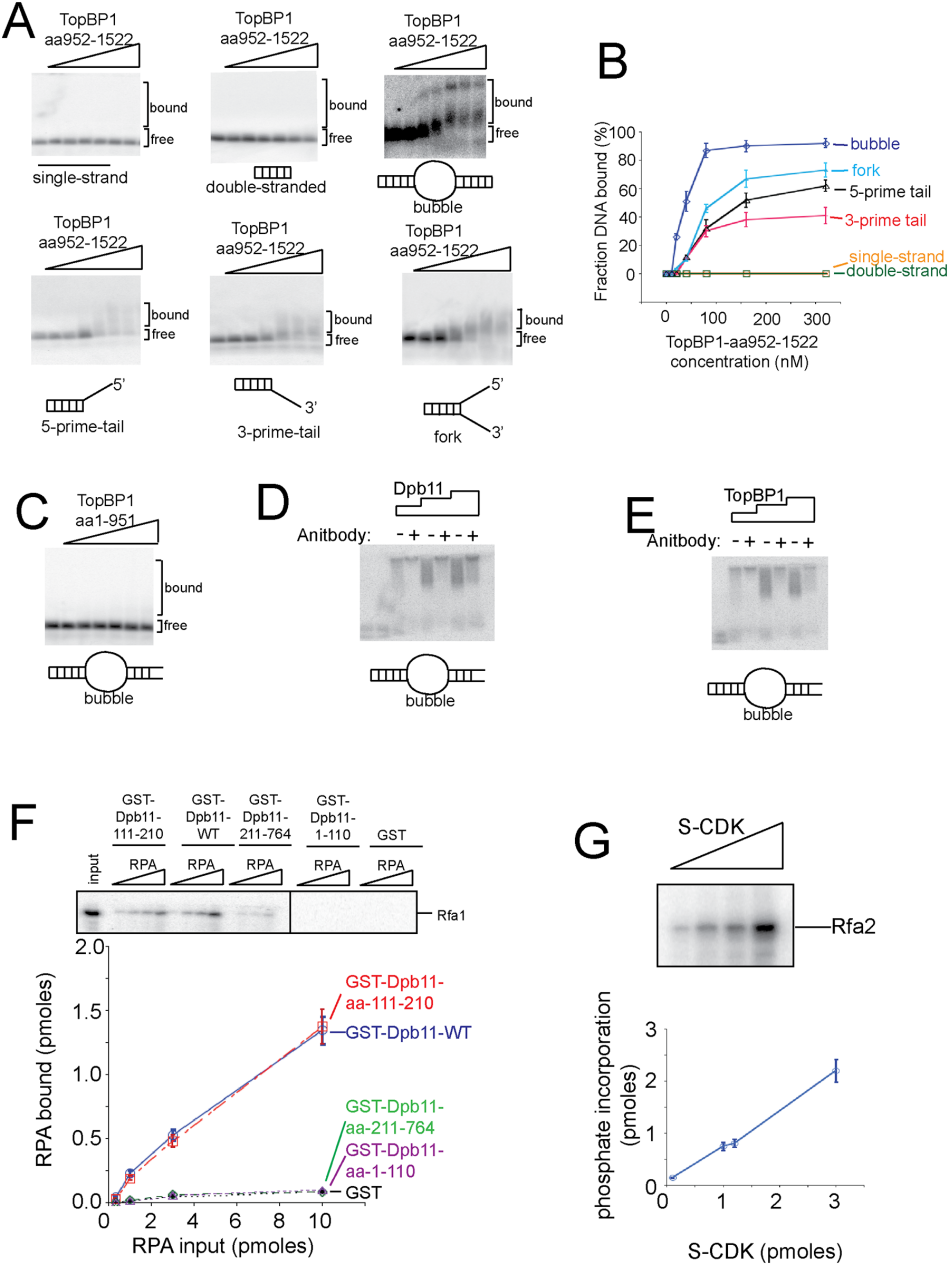
The C-terminal region of TopBP1 binds to branched-DNA structures. (A) Increasing concentrations of the C-terminal region of human TopBP1 (TopBP1aa-952-1522) (0, 10 20, 40, 80, 160, and 320 nM) were incubated with different DNA structures as described in Materials and Methods. The DNA sequences are detailed in Table 1; the DNA was radiolabeled with ^32^P. The products were analyzed by native agarose gel electrophoresis, and the dried gel was exposed to a phosphorimaging screen. (B) Experiments similar to those described in (A) were quantified, averaged, and plotted. The fraction DNA bound was plotted as a function of TopBP1-aa952-1522 concentration. (C) Similar to (A), except the N-terminal region of human TopBP1 was used instead of the C-terminal region. Identical bubble DNA structure and protein concentrations were used as in (A). (D) Similar to 1B, except the experiment was performed in the absence or presence of antibody specific for Dpb11. (E) Similar to 1D, except the experiment was performed in the absence or presence of antibody specific for TopBP1. (F) 10 pmoles of purified GST-Dpb11 (Full-length Dpb11 or fragments of Dpb11) was used to pull-down PKA-RPA. PKA-RPA has an artificial PKA site at the N-terminus of Rfa1 for radiolabeling with ^32^P. The PKA tag is not physiologic; it is used as a means for accurate quantitation. The kinase was removed from the reaction prior to the GST-pulldown experiment. The product of the pulldown was analyzed by SDS/PAGE followed by phosphorimaging. Three independent experiments were quantified, averaged, and plotted. (G) CDK phosphorylation of RPA. 3 pmoles RPA was added to various concentrations of CDK as shown in the figure in the presence of ^32^P-gamma-ATP. The product of the kinase reaction was analyzed by SDS/PAGE followed by phosphorimaging. Three independent experiments were quantified, averaged, and plotted.

1nM radiolabeled DNA was mixed with various concentrations of protein (as detailed in Figs 1 and 2) in 1X EMSA buffer (containing 0.1 mM EDTA, 0.2 mM DTT, 10% glycerol, 40 mg/ml BSA, and 20 mM Tris-HCl, pH 7.5) for 5 minutes at 30°C. 4% Glycerol and 0.01%bromophenol blue were then added to the solution, and the reaction was analyzed by a 0.7% native agarose gel in 0.5 X TBE buffer. The gel was dried and then exposed to a phosphorimaging screen for 1 h. The mean ± standard deviation is shown for three independent experiments.

### Yeast dilutions

Serial dilution was performed as described [32]. Yeast strains in overnight culture (CSM-Ura containing raffinose, 30°C) were transferred into YPGal media containing 50 μg/ml doxycycline and incubated for 2 hrs at 37°C. The 10 fold serial dilution was performed and spotted onto a plate containing CSM-Ura, which was incubated at 30°C (permissive conditions) and a plate containing CSM-Ura+Gal+50μg doxycycline, which was incubated at 37°C (restrictive conditions) for two days.

### Fluorescence activated cell sorting (FACS analysis)

FACS was performed as described [32]. The strains were grown overnight in CSM-Ura media containing raffinose at 30°C. For G_1_ arrest 6x10^6^ cells/ml were treated with α-factor (Zymo Research) for 3 hrs at 37°C in YPGal media containing 50 μg/ml doxycycline. Following extensive washes and addition of 50 μg/ml Pronase (Calbiochem) to fresh YPGal+Doxycycline, cells were further incubated at 37°C. Cells were collected at indicated time intervals and stained with propidium iodide. Cell cycle progression data was obtained using the BD FACS Canto Ruo Special Order System and analyzed using the FACS Diva Software.

### Chromatin immunoprecipitation (ChIP)

For G_1_ arrest and release 6x10^6^ cells/ml were treated with α-factor (Zymo Research) for 3 hrs at 37°C in YPGal media containing 50 μg/ml doxycycline. Following extensive washes and addition of 50 μg/ml Pronase (Calbiochem) to fresh YPGal+Doxycycline, cells were further incubated at 37°C for the indicated time. Chromatin immunoprecipitation was performed as described [33]. We performed PCR with [^32^P-α]-dCTP as a component of the PCR reaction to quantify the amplified product. Formaldehyde cross-linked cells were lysed with glass beads in a Bead Beater. DNA was fragmented by sonication (Branson 450). RPA, Cdc45, or GINS antibody and magnetic protein A beads (Dynabeads protein A, Invitrogen 100.02D) were added to the cleared lysate to immunoprecipitate the DNA. Immunoprecipitates were washed extensively to remove nonspecific DNA. Eluted DNA was then subjected to PCR analysis using primers directed against *ARS305*, *ARS306* or a site midway between *ARS306* and *ARS305* as described [34]. The radioactive band in the native gel, representing specific PCR amplified DNA product was quantified by phosphorimaging and normalized by a reference standard run in the same gel. The reference standard was a PCR reaction with a known quantity of template DNA replacing the immunoprecipitate. The mean ± standard deviation is shown for three independent experiments.

### Co-immunoprecipitation (Co-IP)

For G_1_ arrest and release 6x10^6^ cells/ml were treated with α-factor (Zymo Research) for 3 hrs at 37°C in YPGal media containing 50 μg/ml doxycycline. Following extensive washes and addition of 50 μg/ml Pronase (Calbiochem) to fresh YPGal+Doxycycline, cells were further incubated at 37°C for the indicated time. Co-immunoprecipitation was performed as described [29]. Cells were collected and lysed at 4°C with glass beads in IP buffer (100 mM HEPES-KOH pH 7.9, 100 mM potassium acetate, 10 mM magnesium acetate, 2 mM sodium fluoride, 1 mM PMSF, 0.1 mM Na3VO4, 20 mM β-gycerophosphate, 1% Triton X-100, leupeptin, pepstatin, 1x complete protease inhibitor cocktail without EDTA (Roche)). Lysed material was treated with 200 U of Benzonase nuclease (Novagen 70746–3) at 4°C for 1 hour. Clarified extract was then mixed with 2 μl of specified antibody and rotated for 2 hours at 4°C. Following this, 7 μl of Dynabeads Protein A (Invitrogen 100.01D) beads equilibrated with IP buffer were added to the extract and further rotated for 1 hr at 4°C. Beads were washed twice with 500 μl of IP buffer and finally resuspended in SDS-sample buffer. Western analysis was performed and blots were scanned using the LI-COR Odyssey Infrared Imager and analyzed in the Image Studio 4.0 Software. The mean ± standard deviation is shown for three independent experiments.

### GST-pulldown

The GST-Pulldown assays were performed as described [25]. GST-pulldown reactions were in a volume of 100 μl and contained GST-tagged protein in GST-binding buffer (40 mM Tris-HCl, pH 7.5, 100 mM NaCl, 0.1 mM EDTA, 10% glycerol, 0.1% Triton X-100, 1 mM DTT, 0.7 mg/ml pepstatin, 0.1 mM PMSF, and 0.1 mg/ml BSA) and varying amounts of radiolabeled protein as described in each Fig. Reactions were incubated at 25°C for 1 hour. Following incubation, reactions were added to 40 μl glutathione Sepharose and gently mixed. Binding of GST-tagged protein to the protein was performed for 20 minutes with gentle mixing every two minutes. When the binding was complete, the beads were allowed to settle, the supernatant was removed, and the glutathione beads were washed two times with 0.5 ml GST-binding buffer. After the last wash, 30 μl of 5X SDS sample buffer were added to each reaction, and the samples were heated to 95°C for 10 minutes. For quantitation of every gel, we include an input with a known amount of protein or DNA for quantitation. Samples (20 μ) were then analyzed by SDS-PAGE followed by phosphorimaging and quantitation. The mean ± standard deviation is shown for three independent experiments.

### DNA sequences

DNA sequences are listed in Table 1.

**Table 1:** Related to materials and methods. Oligonucleotides used in this study.

DNA	Sequences
ssDNA	5’ T (TTT)26 T 3’
blunt	5’ ATGTCCTAGCAAGCCAGAATTCGGCAGCGTC 3’ 5’GACGCTGCCGAATTCTGGCTTGCTAGGACAT 3’
3’-tailed	5’ GACGCTGCCGAATTCTGGCTTGCTAGGACAT 3’ 3’ ATGTCCTAGCAAGCCAGAATTCGGCAGCGTCTT (TTT)_26_ 3’
5’-tailed	5’ ATGTCCTAGCAAGCCAGAATTCGGCAGCGTC 3’ 5’ (TTT)_26_ TTGACGCTGCCGAATTCTGGCTTGCTAGGACAT 3’
fork	5’ ATG TCCTAGCAAGCCAGAATTCGGCAGCGTCTT (TTT)_26_ 3’ 5’ (TTT)_26_ TTGACGCTGCCGAATTCTGGCTTGCTAGGACAT 3’
bubble	5’ATGTCCTAGCAAGCCAGAATTCGGCAGCGTCTT(TTT)_26_CCACGTCGGCGTCGCCACGAGC 3’ 5’GCTCGTGGCGACGCCGACGTGGTT(TTT)_26_ GACGCTGCCGAATTCTGGCTTGCTAGGACAT 3’

## Results

### Yeast Dpb11 has high affinity for ssDNA or branched DNA structures, while human TopBP1 has high affinity for branched-DNA structures

We previously found using a GST-pulldown assay that Dpb11 binds directly to single-stranded DNA (ssDNA), while the interaction between GST-Dpb11 and double-stranded DNA (dsDNA) is at background levels [23]. We postulated that Dpb11 might bind to origin DNA as it is melted during replication initiation. The DNA structure that is found at an origin during melting is branched. We used the electrophoretic mobility shift assay (EMSA) with native agarose gel to assess the interaction between Dpb11 and various DNA structures to determine the influence of DNA-branching on Dpb11 binding (Fig 1B and 1C). We found that 40 nM Dpb11 is sufficient to bind more than half of ssDNA, whereas 320 nM Dpb11 is required to bind more than half of dsDNA, confirming the previous results of our GST-pulldown assay (Fig 1B and 1C). Using EMSA, we found that 20 nM Dpb11 binds to nearly 80% of bubble-shaped DNA and forked-shaped DNA, (Fig 1B and 1C), while 80 nM Dpb11 is required to bind more than half of duplex DNA with a single 3’–single-strand extension or a 5’–single-strand extension (Fig 1B and 1C). These data suggest that Dpb11 binds tightly to single-stranded DNA and also branched DNA structures.

The human homolog of Dpb11 is TopBP1, thus we next determined whether the high affinity of Dpb11 for branched DNA structures is conserved for human TopBP1 (Fig 1D and 1E). We found that human TopBP1, unlike Dpb11, cannot shift 50% or more of single-stranded DNA or double-stranded DNA at concentrations up to 320 nM (Fig 1D and 1E). These results are consistent with other reports suggesting that TopBP1, unlike Dpb11, binds weakly to unmodified ssDNA or dsDNA (Fig 1D and 1E) [26]. However, when we examined branched DNA structures, we found that human TopBP1 at a concentration of only 20 nM shifts nearly 50% of bubble-shaped DNA, forked DNA, or double-stranded DNA with a 5’ or 3’ single-stranded extension (Fig 1D and 1E). These data suggest that TopBP1 has a specific affinity for branched-shaped DNA structures. These data suggest that the ssDNA binding properties of Dpb11 are not conserved in human TopBP1, but tight binding to bubble- or forked-shaped DNA is observed for human TopBP1 and yeast Dpb11. We next identified the C-terminal region of TopBP1 by sequence conservation with Dpb11 using the ClustalW protein alignment software. We then found that the C-terminal region of TopBP1 (amino acids 952–1522) binds to branched-DNA (Fig 2A and 2B), while the N-terminal region of human TopBP1 has no affinity for bubble-shaped DNA (Fig 2C). The C-terminal region of TopBP1 binds to bubble-shaped DNA with higher specificity compared to full-length TopBP1.

We performed electrophoretic mobility shift experiments with Dpb11 and bubble-shaped DNA in the absence and presence of antibody specific for Dpb11, and we observe a supershift in the presence of antibody, confirming that it is a Dpb11-DNA complex (Fig 2D). We also performed a similar experiment with human TopBP1 with antibodies specific for TopBP1, and we observed a supershift as well, confirming that is a TopBP1-DNA complex (Fig 2E).

**Fig 3:**
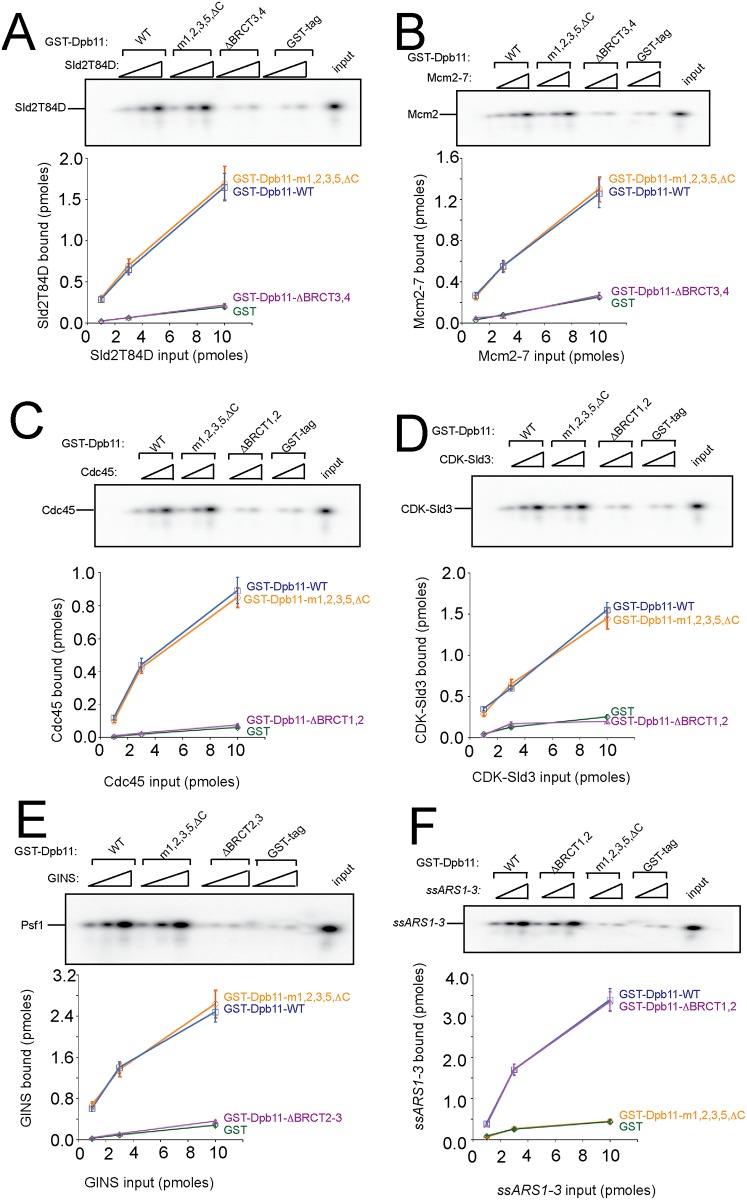
Dpb11-m1,2,3,5, *Δ*C binds to known cellular binding partners like wild-type Dpb11. **Purified proteins were used in these experiments.** (A) 10 pmoles of purified GST-Dpb11 (GST-Dpb11-m1,2,3,5*Δ*C, GST-Dpb11-wild-type, GST-Dpb11-*Δ*BRCT3,4, or GST-tag) was used to pull-down PKA-Sld2T84D. PKA-Sld2T84D has an artificial PKA site at the N-terminus for radiolabeling with ^32^P. The PKA tag is not physiologic; it is used as a means for accurate quantitation. The kinase was removed from the reaction prior to the GST-pulldown experiment. The product of the pulldown was analyzed by SDS/PAGE followed by phosphorimaging. Three independent experiments were quantified, averaged, and plotted. (B) Similar to (A), except Mcm2-7 was used in place of Sld2T84D. The Mcm2-7 complex bears a PKA tag at the N-terminus of Mcm2. (C) Similar to (A), except Cdc45 was used in place of Sld2T84D, and GST-Dpb11-*Δ*BRCT1,2 was used in place of GST-Dpb11-*Δ*BRCT3,4 as a negative control (D) Similar to (C), except CDK-phosphorylated Sld2 was used in place of Cdc45. (E) Similar to (A) except GINS was used in place of Sld2T84D, and GST-Dpb11-*Δ*BRCT2-3 was used in place of GST-Dpb11-*Δ*BRCT3,4 as a negative control. GST-Dpb11-*Δ*BRCT2-3 lacks the region of Dpb11 between the BRCT2 and BRCT3 motifs (GINS-binding region). The GINS complex bears a PKA tag at the N-terminus of Psf1. (F) Similar to (A), except *ssARS1-1* was used in place of Sld2T84D, and GST-Dpb11-*Δ*BRCT1,2 was used as a positive control.

We previously described a mutant of Dpb11 that does not bind single-stranded DNA using the GST-pulldown assay (Dpb11-m1,2,3,5-*Δ*C) [23]. We next examined whether this mutant binds to branched DNA structures using the EMSA, and found that concentrations up to 320 nM of Dpb11-m1,2,3,5-*Δ*C does not shift ssDNA, dsDNA, bubble-shaped DNA, or forked-shaped DNA (Fig 1F). Since we also found that Dpb11-m1,2,3,5-*Δ*C binds to Sld3, Sld2, Mcm2-7, CDC45, and GINS like wild-type Dpb11 ([23] and Fig 3), we have identified a mutant of Dpb11 that is specifically defective for DNA binding.

### Dpb11 binds stably to CDK-phosphorylated RPA in the presence of bubble-shaped DNA

**Fig 4:**
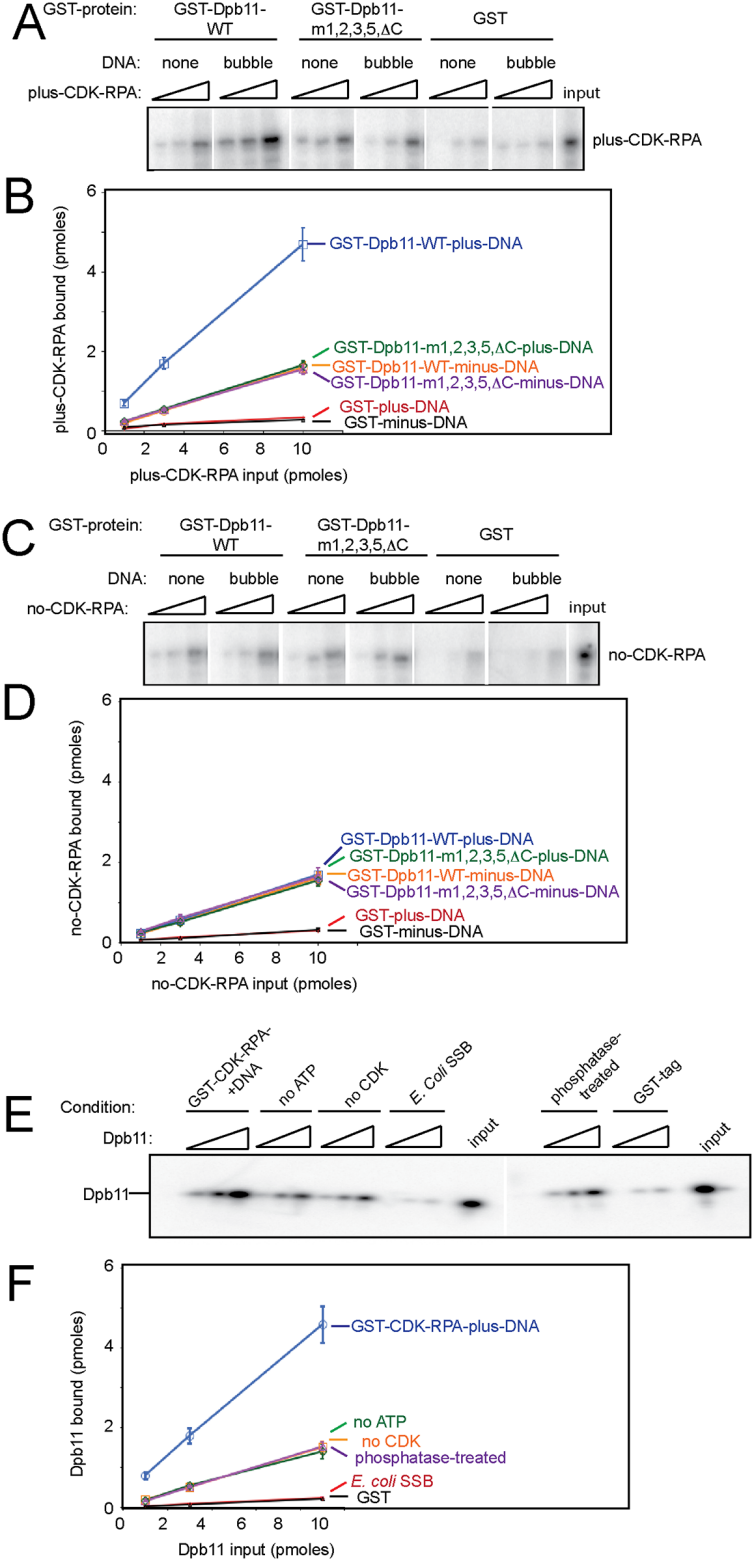
Dpb11 binds stably to CDK-phosphorylated RPA in the presence of bubble-shaped DNA. (A,B) 10 pmoles of purified GST-Dpb11 (GST-Dpb11-m1,2,3,5*Δ*C, GST-Dpb11-wild-type, or GST-tag) was used to pull-down CDK-phosphorylated RPA in the absence or presence of 10 pmol bubble-shaped DNA. RPA has an artificial PKA site at the N-terminus of Rfa1 for radiolabeling with ^32^P. The PKA tag is not physiologic; it is used as a means for accurate quantitation. The kinase was removed from the reaction prior to the GST-pulldown experiment. The product of the pulldown was analyzed by SDS/PAGE followed by phosphorimaging. (B) Experiments similar to (A) were quantified, averaged, and plotted. (C) Similar to (A), except the RPA was not phosphorylated by CDK. (D) Experiments similar to (C) were quantified, averaged, and plotted. (E) Similar to (A), except GST-RPA was used to pulldown radiolabeled Dpb11. (F) Experiments similar to (e) were quantified, averaged, and plotted.

During the process of origin melting, the single-stranded DNA that is generated is ultimately bound by RPA, the eukaryotic single-stranded DNA binding protein. We considered that Dpb11 may contribute to origin melting by functioning with RPA to stabilize melted origin ssDNA. We found first that RPA binds directly to the BRCT2 domain of Dbp11 using the GST-pulldown assay (Fig 2F). To determine whether Dpb11 functions with RPA and melted DNA, we incubated GST-Dpb11 with CDK-phosphorylated RPA and performed a quantitative GST-pulldown assay in the absence or presence of bubble-shaped DNA (Fig 4A and 4B). In this assay, the Rfa1 subunit bears an artificial PKA tag for radiolabeling with ^32^P, and purified S-CDK is incubated with RPA and ATP to achieve phosphorylation (Fig 2G). We found that GST-Dpb11-wild-type pulled down a substantial fraction (approximately 50%) of CDK-phosphorylated RPA in the presence of bubble-shaped DNA. In the absence of DNA, the pulldown efficiency was substantially reduced to approximately 15% (Fig 4A and 4B). These data suggest that bubble-shaped DNA substantially stimulates the interaction between Dpb11 and RPA. Similar results were observed when single-stranded DNA was substituted for bubble-shaped DNA (approximately 50% pulldown efficiency in either case, not shown). Interestingly, the Dpb11 mutant that is specifically defective for binding DNA, (Dpb11-m1,2,3,4,*Δ*C), did not bind exhibit DNA-induced stimulation of RPA binding (approximately 15% pulldown efficiency in the absence or presence of bubble-shaped DNA). These data suggest that Dpb11 interaction with DNA is required for maximal interaction between Dpb11 and RPA. As an additional control, we repeated the assay with RPA that was not phosphorylated by CDK (Fig 4C and 4D). This RPA protein lacking S-CDK phosphorylation exhibited no increase in GST-Dpb11 binding in response to the addition of bubble-shaped DNA (15% pulldown efficiency in the absence or presence of DNA). These data suggest that S-CDK phosphorylation of RPA and melted DNA interaction with Dpb11 are required for maximal interaction between Dpb11 and RPA.

We also performed the reverse pulldown, with GST-RPA pulling down radiolabeled Dpb11, and we found the same stimulatory effect of CDK and DNA on the pulldown efficiency (Fig 4E and 4F). These data suggest that PKA phosphorylation of RPA is not responsible for the inhibition observed, since RPA is not phosphorylated by PKA in this assay. We also performed additional control experiments. We replace RPA with *E*. *coli* SSB (Single-stranded-DNA binding protein), and observe no interaction with Dpb11. We also found that the omission of ATP from the kinase reaction, or the addition of Lambda protein phosphatase after the kinase reaction, eliminates the stimulatory effect of CDK on the pulldown efficiency (Fig 4E and 4F). These data suggest that the most efficient pulldown between RPA and Dpb11 occurs when RPA is phosphorylated by CDK, and bubble-shaped DNA is present in the reaction.

### Cells expressing *dpb11-m1*,*2*,*3*,*5*,*ΔC* exhibit a defect in cell growth and DNA replication

**Fig 5:**
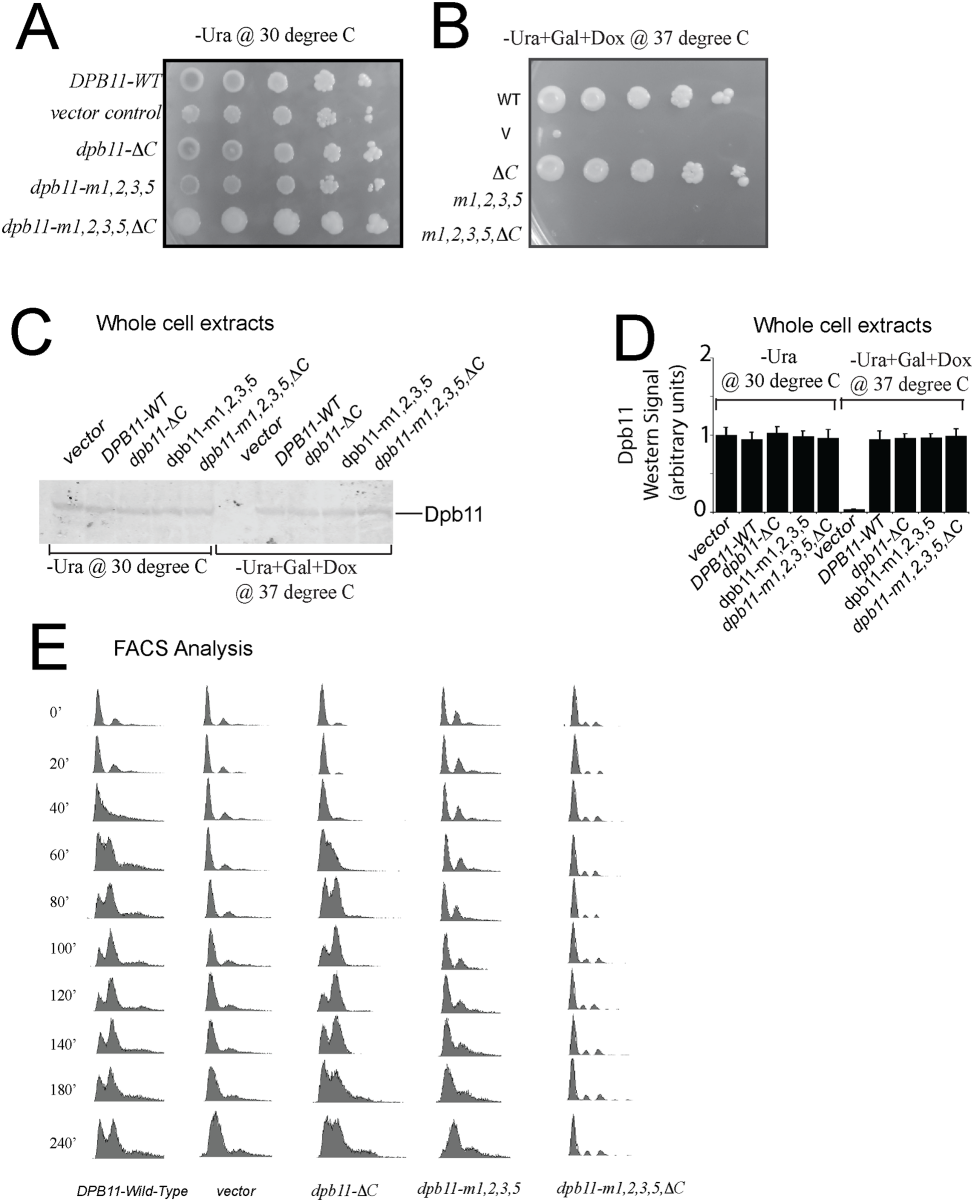
Cells expressing *dpb11-m1*,*2*,*3*,*5*,*ΔC* exhibit a defect in cell growth and DNA replication. (A,B) Ten-fold serial dilutions of *dpb11-td* cells expressing *DPB11*-wild type, vector only control, *dpb11-ΔC*, *dpb11-m1*,*2*,*3*,*5*, or *dpb11-m1*,*2*,*3*,*5*,*ΔC* at the (A) permissive conditions (CSM-Ura, 30°C) or (B) restrictive conditions (CSM-Ura+gal+doxycycline, 37°C). (C) Western analysis of whole cell extracts of *dpb11-td* cells expressing vector, *DPB11*-wild type, *dpb11-ΔC*, *dpb11-m1*,*2*,*3*,*5*, or *dpb11-m1*,*2*,*3*,*5*,*ΔC* under restrictive conditions. (D) Experiments similar to those in (C) were averaged and plotted. Bands from Western experiments were quantified with Odyssey software as described in Materials and Methods. (E) FACS analysis was performed as described in Materials and Methods on *dpb11-td* cells expressing *DPB11*-wild type, vector only control, *dpb11-ΔC*, *dpb11-m1*,*2*,*3*,*5*, or *dpb11-m1*,*2*,*3*,*5*,*ΔC* under restrictive conditions. Cells were synchronized in G_1_ with s were synchronized in Gl, D) Experiments similα-factor for the time points indicated.

We next investigated whether Dpb11-DNA interaction is required for DNA replication and cell growth. To accomplish this, we used a previously published *dpb11-td* degron strain [23, 35]. This strain results in complete degradation of the native Dpb11 protein under restrictive conditions (+Galactose, +Doxycycline, 37°C). We transformed these cells with plasmid expressing wild-type *DPB11*, empty vector, or mutant *dpb11* (*dpb11-m1*,*2*,*3*,*5*, *dpb11-ΔC*, or *dpb11-m1*,*2*,*3*,*5*, *ΔC*) under control by native promoter. We found that under permissive conditions (-Galactose,-Doxycycline 30°C), there is equal growth of cells transformed with wild-type *DPB11*, empty vector, or mutant *dpb11* (Fig 5A). However, under restrictive conditions, we found that cells with empty vector or vector expressing *dpb11-m1*,*2*,*3*,*5* or *dpb11-m1*,*2*,*3*,*5*,*ΔC* exhibit a severe growth defect compared to cells expressing wild-type *DPB11* or *dpb11-ΔC* (Fig 5B).

Western analysis of whole cell extracts reveals equivalent expression of wild-type or mutant Dpb11 under permissive or restrictive conditions, and loss of Dpb11 expression for cells harboring empty vector under restrictive conditions (Fig 5C and 5D). Taken together, these data suggest that Dpb11-binding to DNA is required for cell growth. We also examined DNA replication in these cells under restrictive conditions by FACS analysis, and found that cells with empty vector or vector expressing *dpb11-m1*,*2*,*3*,*5* or *dpb11-m1*,*2*,*3*,*5*,*ΔC* exhibit a severe DNA replication defect compared to cells expressing wild-type *DPB11* or *dpb11-ΔC* (Fig 5E). These data suggest that Dpb11 interaction with DNA is required for DNA replication.

### Cells expressing *dpb11-m1*,*2*,*3*,*5*, *ΔC* exhibit a severe defect in RPA and GINS chromatin immunoprecipitation signal using primers directed at early replication origins

**Fig 6:**
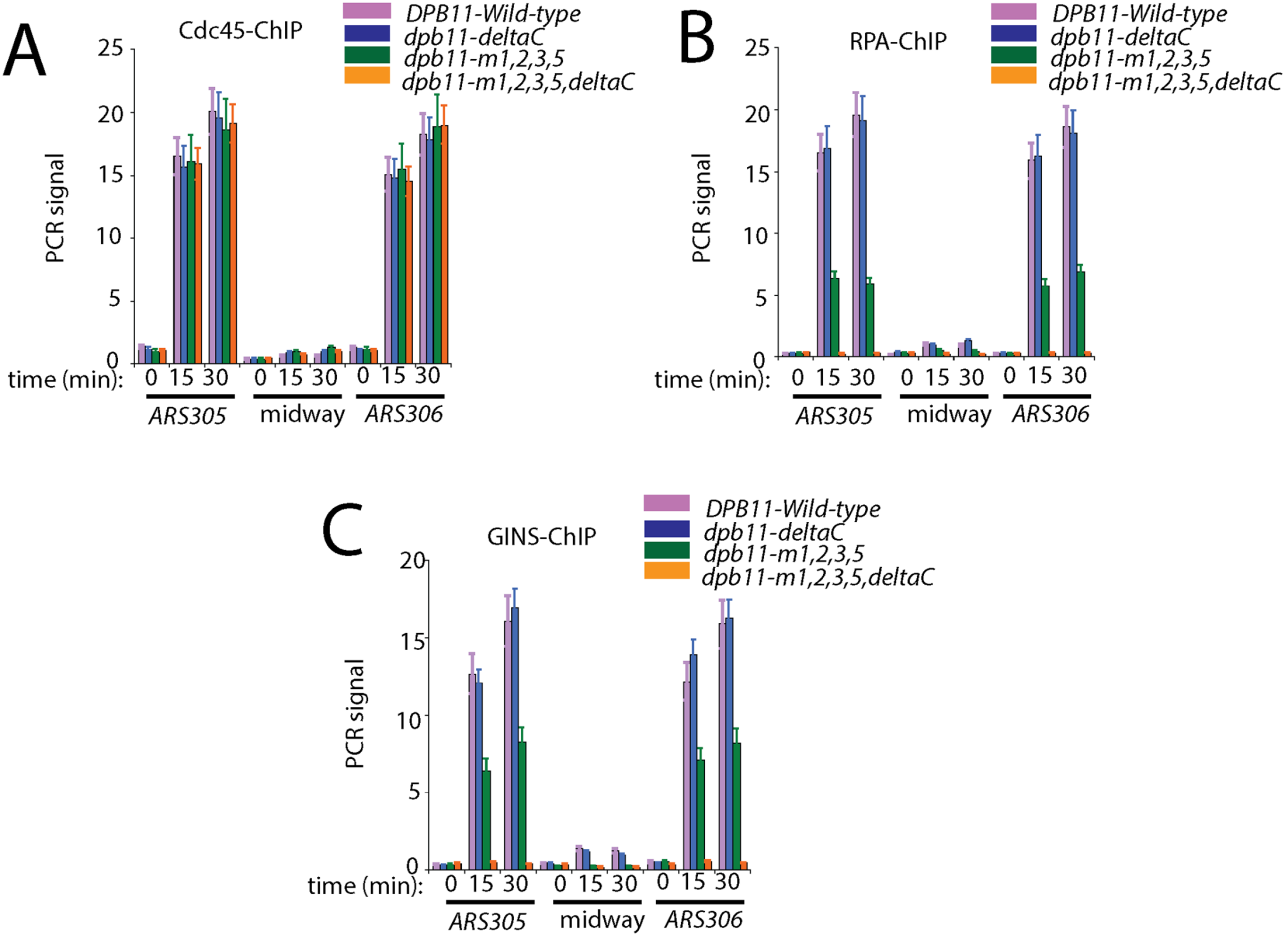
Cells expressing *dpb11-m1*,*2*,*3*,*5*,*ΔC* exhibit a defect in severe defect in RPA and GINS chromatin immunoprecipitation signal using primers directed at early replication origins. (A,B,C) Chromatin immunoprecipitation was performed as described in Materials and Methods. *dpb11-td* cells under restrictive conditions, harboring plasmid expressing wild-type *DPB11* or mutant *dpb11* as indicated, were arrested with α-factor and then released into medium lacking α-factor for the time points indicated. PCR primers were used that target the early yeast origin *ARS305*, *ARS306* or a region positioned midway between *ARS305* and *ARS306*. [^32^P-[306region positioned midway between PCR primers were used that target the early yeast origin mers directed at early replication origins.e GINS-ChIP signal at early replica used for the chromatin immunoprecipitation experiment were targeting Cdc45 (A), RPA (B), or GINS (C), as described in Materials and Methods.

We next examined the role of Dpb11 interaction with DNA in DNA replication using ChIP assays (Fig 6). Cdc45 interaction with early origin DNA was normal for cells expressing *dpb11-m1*,*2*,*3*,*5*,*ΔC* under restrictive conditions, suggesting that Dpb11-DNA interaction is not required for Cdc45 recruitment to origins, as expected (Fig 6A). The RPA-ChIP signal was substantially decreased for cells expressing *dpb11-m1*,*2*,*3*,*5*,*ΔC* compared to wild-type (Fig 6B). It is interesting to observe that the RPA-ChIP signal for cells expressing *dpb11-m1*,*2*,*3*,*5*,*ΔC* is lower than that of *dpb11-m1*,*2*,*3*,*5* (Fig 6B). These data suggest that when Dpb11 interaction with DNA is specifically disrupted, there is no recruitment of RPA to origins. The GINS-ChIP signal at early replication origins is also substantially decreased for cells expressing *dpb11-m1*,*2*,*3*,*5*,*ΔC* compared to wild-type. This effect is likely to be indirect, since Dpb11-GINS interaction *in vitro* is like wild-type for the *dpb11-m1*,*2*,*3*,*5*,*ΔC* mutant (Fig 3).

### Cells expressing *dpb11-m1*,*2*,*3*,*5*,*ΔC* exhibit a severe defect in RPA and GINS attachment to Mcm2

**Fig 7:**
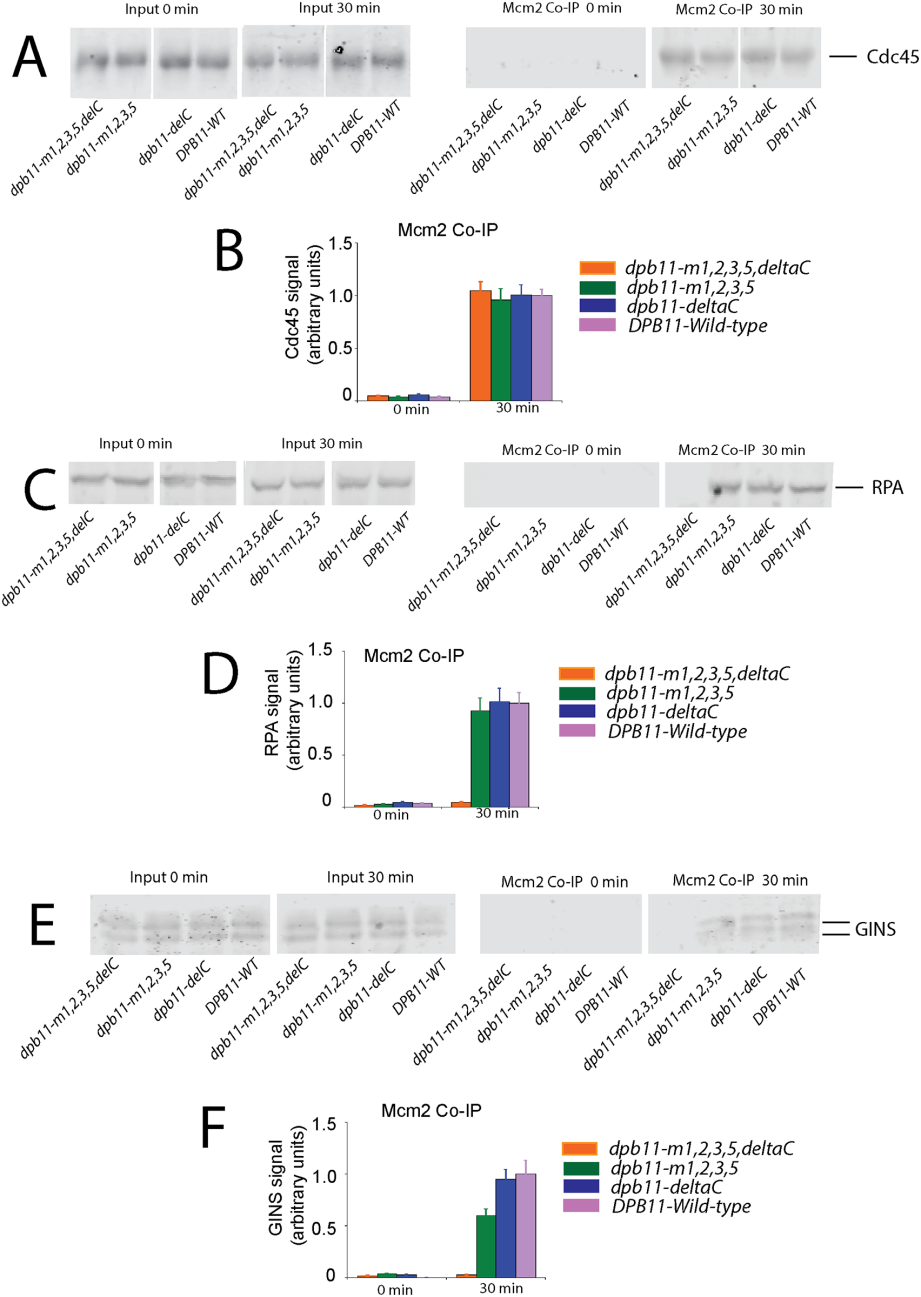
Cells expressing *dpb11-m1*,*2*,*3*,*5*,*ΔC* exhibit a defect in severe defect in RPA and GINS attachment to Mcm2. (A-F) Co-immunoprecipitation was performed as described in Materials and Methods. *dpb11-td* cells under restrictive conditions, harboring plasmid expressing wild-type *DPB11* or mutant *dpb11* as indicated, were arrested with α-factor and then released into medium lacking α-factor for the time points indicated. Whole cell extracts (input), or extracts precipitated with antibodies directed against Mcm2 (Mcm2 Co-IP), were analyzed by Western analysis for Cdc45 (A), RPA (C), or GINS (E). Mcm2 Co-IP results from repeat Western experiments similar to (A, C and E) were quantified and plotted for Cdc45 (B), RPA (D), or GINS (F).

We then investigated whether Dpb11 interaction with DNA is required for Cdc45, RPA, or GINS interaction with Mcm2 *in vivo* (Fig 7). Cdc45-Mcm2 interaction is like wild-type for cells expressing *dpb11-m1*,*2*,*3*,*5*,*ΔC*, suggesting that Dpb11 interaction with DNA is not required for Cdc45 attachment to Mcm2-7 (Fig 7A and 7B). However, RPA-Mcm2 interaction (Fig 7C and 7D) and GINS-Mcm2 interaction (Fig 7E and 7F) is substantially reduced for cells expressing *dpb11-m1*,*2*,*3*,*5*,*ΔC*. These data suggest that Dpb11 interaction with DNA is required for RPA and GINS recruitment to Mcm2-7 *in vivo*.

In summary, we compared the consequences of expressing *dpb11-m1*,*2*,*3*,*5*, *dpb11-ΔC*, *dpb11-m1*,*2*,*3*,*5*,*ΔC*, or *DPB11-WT* in a *dpb11-td* cell under restrictive conditions. For cell growth and DNA replication, *dpb11-m1*,*2*,*3*,*5* and *dpb11-m1*,*2*,*3*,*5*,*ΔC* exhibited severe growth and DNA replication defects compared to *dpb11-ΔC* or *DPB11-WT* (Fig 5). By ChIP analysis, *dpb11-m1*,*2*,*3*,*5* yielded a modest decrease in RPA and GINS recruitment to origins compared to *DPB11-WT*, while *dpb11-m1*,*2*,*3*,*5*,*ΔC* exhibited a severe defect in RPA and GINS recruitment to origins compared to *DPB11-WT* (Fig 6). On the other hand, using Co-IP analysis, we found that *dpb11-m1*,*2*,*3*,*5*,*ΔC* exhibited a severe defect in Mcm2-GINS or Mcm2-RPA interaction compared to *DPB11-WT* (Fig 7), while *dpb11-m1*,*2*,*3*,*5* exhibited no defect compared to *DPB11-WT*. Thus, there is a difference between the results of the ChIP compared to the Co-IP analysis for *dpb11-m1*,*2*,*3*,*5*, since we observe modest defects in GINS and RPA association with origins using ChIP, while we observe no defects in GINS or RPA association with Mcm2 using Co-IP, consistent with our previous findings [23]. Although we do not know the reason for these differences, the difference between the results may be related to the fact that ChIP monitors proteins associated with origins, while Mcm2-Co-IP directly examines Mcm2-protein interaction; thus, the techniques are not the same. For the DNA-binding defective mutant of Dpb11, *dpb11-m1*,*2*,*3*,*5*,*ΔC*, we observe severe RPA-ChIP and GINS-ChIP defects, and severe Mcm2-GINS and Mcm2-RPA Co-IP defects. Thus, the *dpb11-m1*,*2*,*3*,*5*,*ΔC* mutant is severely defective for GINS and RPA recruitment to origins as measured by ChIP, and severely defective for GINS and RPA association with Mcm2 by Co-IP.

## Discussion

### Budding yeast Dpb11 and human TopBP1 bind tightly to branched-DNA structures

Using purified proteins, we found that budding yeast Dpb11 binds tightly to single-stranded DNA, bubble-shaped DNA, or forked-DNA structures. The human homolog of Dpb11, TopBP1, also binds tightly to bubble-shaped or forked-DNA structures, while TopBP1 binds weakly to single-stranded DNA or double-stranded DNA. Thus, the high affinity of Dpb11 for branched DNA structures is also observed for human TopBP1. We also characterized a mutant of Dpb11, (Dpb11-m1,2,3,5,*Δ*C, or Dpb11-K463E, K469E, R538E,K549E, *Δ*aa616-764), that is specifically defective for binding to DNA. This mutant behaves like wild-type for binding Cdc45, Mcm2-7, Sld2, Sld3 and GINS, suggesting that the defect in DNA binding is specific.

### Dpb11 interacts stably with CDK-phosphorylated RPA in the presence of bubble-shaped DNA

We next studied the interaction affinity between Dpb11 and RPA using a GST-pulldown assay. We found that GST-Dpb11 pulls down ~ 50% of CDK-phosphorylated-RPA in the presence of bubble-shaped DNA. In the absence of DNA, the pulldown efficiency decreases to 15%. Furthermore, the pulldown efficiency in the presence of DNA is reduced to 15% with GST-Dpb11-m1,2,3,5,*Δ*C, or with the addition of RPA that is not phosphorylated by S-CDK. These data suggest that S-CDK phosphorylation of RPA and Dpb11 interaction with melted DNA are required for maximal interaction between Dpb11 and RPA. These data suggest a cellular model wherein during S phase, Dpb11 binds to melted origin DNA, and Dpb11 bound to melted DNA recruits S-CDK phosphorylated RPA to the origin. We cannot rule out that RPA interaction with melted origin DNA *in vivo* can occur without Dpb11, since RPA binds tightly to single-stranded DNA on its own.

### Dpb11 interaction with DNA may be required for origin melting in budding yeast cells

We next studied the effect of expressing wild-type levels of a *dpb11* mutant specifically defective for binding DNA in the absence of wild-type *DPB11*, and found a substantial decrease in GINS and RPA recruitment to replication origins and Mcm2. Cdc45 recruitment to replication origins was normal in these cells. The substantial decrease in RPA recruitment to replication origins and Mcm2 may reflect a defect in origin melting, resulting from a specific defect in Dpb11 for DNA interaction. Since our *in vitro* data demonstrate a high affinity of Dpb11 for single-stranded DNA and branched-DNA structures that mimic a partially melted replication origin, we propose that Dpb11 may bind to branched-DNA structures at a replication origin to stabilize the partially melted DNA during S phase. Furthermore, since Dpb11 binds stably to CDK-phosphorylated RPA in the presence of bubble-shaped DNA, we speculate that Dpb11 may function with RPA to further stabilize the melted state of the DNA.

Other models for origin melting *in vivo* are also consistent with our data. For example, origin melting may occur in the absence of Dpb11, and Dpb11-DNA interaction may be required for some other function in DNA replication initiation, such as helicase activation. Furthermore, RPA may bind to melted origin DNA prior to Dpb11 interaction with RPA, since we cannot dissect the sequence of events for origin melting *in vivo*. Further exploration of origin melting with an *in vitro* reconstitution system may help distinguish between these two models.

### A model for origin melting in eukaryotes

**Fig 8:**
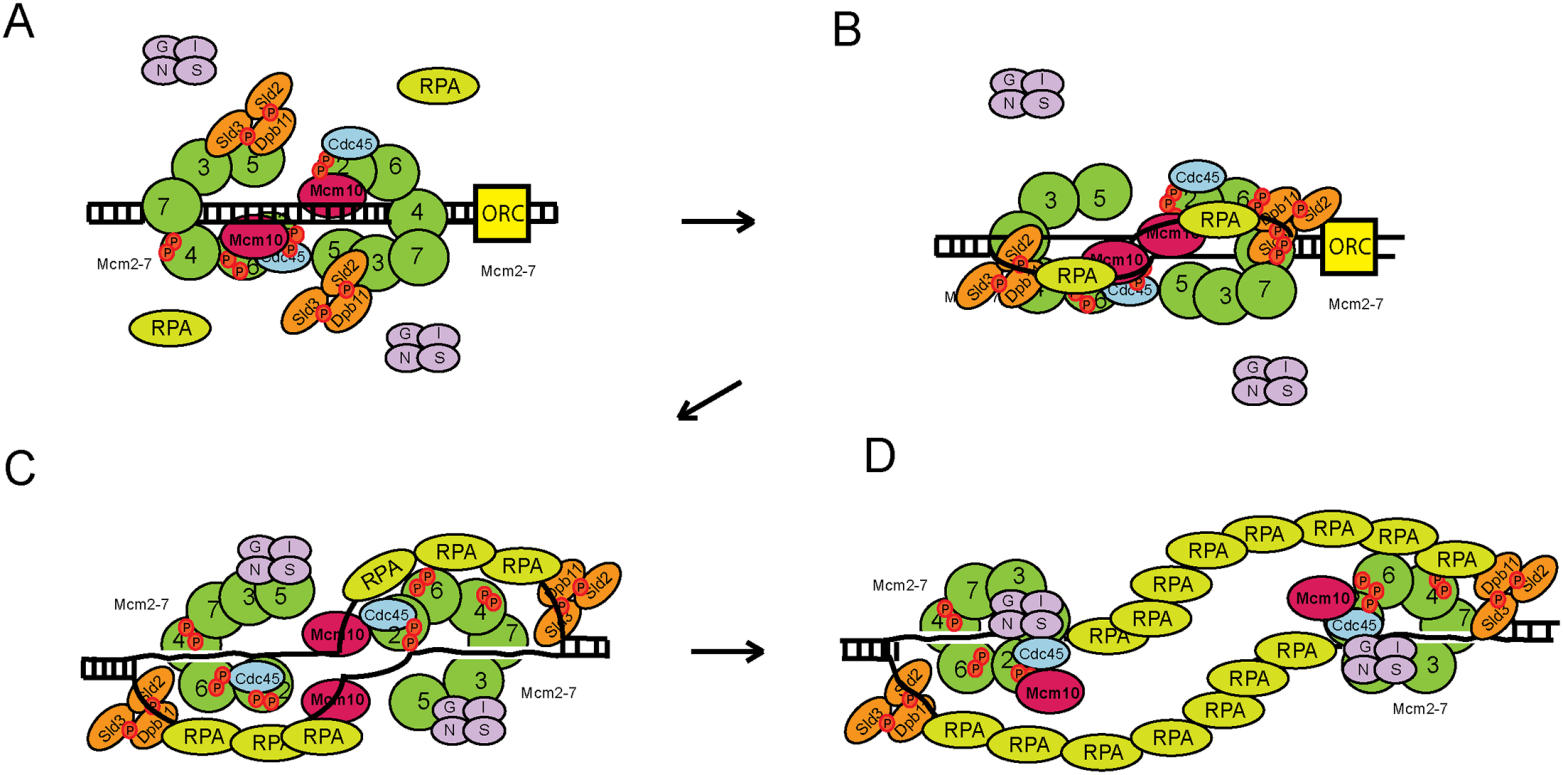
Model for the initiation of DNA replication in budding yeast. (A) Mcm2-7 loads as a double hexamer to encircle double-stranded origin DNA during late M and G_1_ phases. During S phase, the Mcm2-7 ring opens at the Mcm2-Mcm5 interface. Sld2, Sld3, and Dpb11 block the interaction between GINS and Mcm2-7. (B) Dpb11, Sld3, Sld2, and Mcm10 bind to the melted DNA strand as it is extruded from the central channel of Mcm2-7. Dpb11 recruits RPA to the melted DNA. (C) RPA and the replication initiation factors stabilize the melted DNA strand. Sld2-Sld3-Dpb11 binds to melted origin DNA, dissociating from Mcm2-7 and allowing GINS binds to Mcm3/Mcm5 by a passive sequestration mechanism. (D) The CMG is assembled, and bidirectional replication fork unwinding is initiated.

In *Escherichia coli*, the ATPase and DNA binding activity of DnaA enables this protein to melt origin DNA [36]. DnaC acts upon the melted origin DNA in *E*. *coli* to load the replication helicase DnaB to encircle single-stranded DNA [37]. In the eukaryotic viral replication systems, the SV40 large T antigen or the papilloma E1 helicase accomplishes origin melting [38–40]. These viral helicases bind to RPA to hand-off the melted origin DNA to RPA [41]. We speculate that for the eukaryotic cellular helicases, Mcm2-7 and/or ORC melt(s) the origin DNA, since Mcm2-7 and ORC possess ATPase activity [10, 42, 43]. Dpb11 may function with the Mcm2-7 proteins, since this protein binds to Mcm2-7 and melted DNA, to prevent DNA reannealing during the process of origin melting [23, 28, 30, 44]. Dpb11 may also function with RPA to stabilize melted origin DNA, since Dpb11 and RPA form a complex with melted DNA (Fig 8). Other replication initiation proteins may participate in the origin melting process as well, since Mcm10 [44–46], Sld2 [28, 29], and Sld3 [30, 47] also bind directly to single-stranded DNA. Thus, we speculate that for eukaryotes, while the Mcm2-7 or ORC proteins may melt the origin because these proteins possess ATPase activity, Dpb11 and other initiation proteins may function with RPA to stabilize the melted origin DNA (Fig 8). It is also possible that RPA binds to melted origin DNA without requiring Dpb11*in vivo*, since RPA alone has high affinity for single-stranded DNA. In this alternative model, Dpb11 binds to the RPA-DNA complex after the origin is melted, and Dpb11-DNA interaction is then required for some other function during replication initiation, such as helicase activation.

### A model for GINS recruitment to Mcm2-7 in eukaryotes

The lack of GINS recruitment to Mcm2-7 in cells expressing *dpb11-m1*,*2*,*3*,*5ΔC* is likely indirect, since Dpb11-m1,2,3,5*Δ*C binds to GINS like wild-type Dpb11. We previously found that GINS binds stably and directly to purified Mcm2-7 *in vitro* [27]. Sld2, Sld3, and Dpb11 compete with GINS for binding to Mcm2-7 [23, 27, 29, 32]. However, in the presence of single-stranded DNA, the ssDNA sequesters Sld2, Sld3, and Dpb11 from Mcm2-7, allowing GINS to bind to Mcm2-7 by a passive mechanism [23, 29, 47] (Fig 8). This data is consistent with work in this manuscript, and we speculate that the defect in GINS attachment to Mcm2-7 is secondary to the defect in origin melting.

### A conserved mechanism for origin melting among eukaryotes?

A recent report demonstrates that Xenopus TopBP1 binds to RPA that is bound to single-stranded DNA, and TopBP1-RPA-ssDNA interaction is required for DNA repair [48]. However, a comparison between yeast and Xenopus systems reveals significant differences. For example, Dpb11 binds directly to single-stranded DNA, while Xenopus TopBP1 does not bind to single-stranded DNA in the absence of RPA. Furthermore, in budding yeast, Dpb11-DNA interaction is required for DNA replication, while for Xenopus the interaction between TopBP1 and RPA-coated ssDNA is required for DNA repair [48]. We speculate that for Xenopus and humans, TopBP1 may bind directly to branched-DNA to recruit RPA to replication origins, based upon work reported here. In the future it will be interesting to determine whether TopBP1 interaction with melted DNA and RPA is required for origin melting in Xenopus and humans.
